# A method for calling copy number polymorphism using haplotypes

**DOI:** 10.3389/fgene.2013.00165

**Published:** 2013-09-23

**Authors:** Gun Ho Jang, Jason D. Christie, Rui Feng

**Affiliations:** Department of Biostatistics and Epidemiology, Center for Clinical Epidemiology and Biostatistics, University of PennsylvaniaPhiladelphia, PA, USA

**Keywords:** CNV, CNP, GWAS, haplotype, joint SNP and CNV calling, integrated SNP and CNV

## Abstract

Single nucleotide polymorphism (SNP) and copy number variation (CNV) are both widespread characteristic of the human genome, but are often called separately on common genotyping platforms. To capture integrated SNP and CNV information, methods have been developed for calling allelic specific copy numbers or so called copy number polymorphism (CNP), using limited inter-marker correlation. In this paper, we proposed a haplotype-based maximum likelihood method to call CNP, which takes advantage of the valuable multi-locus linkage disequilibrium (LD) information in the population. We also developed a computationally efficient algorithm to estimate haplotype frequencies and optimize individual CNP calls iteratively, even at presence of missing data. Through simulations, we demonstrated our model is more sensitive and accurate in detecting various CNV regions, compared with commonly-used CNV calling methods including PennCNV, another hidden Markov model (HMM) using CNP, a scan statistic, segCNV, and cnvHap. Our method often performs better in the regions with higher LD, in longer CNV regions, and in common CNV than the opposite. We implemented our method on the genotypes of 90 HapMap CEU samples and 23 patients with acute lung injury (ALI). For each ALI patient the genotyping was performed twice. The CNPs from our method show good consistency and accuracy comparable to others.

## 1. Introduction

DNA copy number variation (CNV) refers to differences in genomic DNA with varying numbers of gene copies including segmental amplification, deletion, and loss of heterozygosity. CNVs are found widespread in the human genome, covering approximately 18% of the genome (Freeman et al., 2006; Redon et al., 2006; McCarroll and Altshuler, 2007; Database of Genomic Variates). Increasing evidence shows that CNVs accounts for a significant portion of phenotypic variation (Iafrate et al., 2004; Sebat et al., 2004; Tuzun et al., 2005; Conrad et al., 2006; McCarroll et al., 2006; Redon et al., 2006) yet are far underestimated for human diseases and conditions (Sebat et al., 2007). A comprehensive study suggested that the total amount of sequence variation involving CNVs between two healthy subjects was actually higher than that for Single nucleotide polymorphisms (SNPs) (Redon et al., 2006), which was supported by the increasing number and resolution of CNV discoveries (Korbel et al., 2007). A systematic evaluation of five widely used array-based CNV detection programs suggested that existing methods have conservative sensitivity in CNV detection.

Recently, high-density SNP genotyping arrays have gained substantial attention for CNV detection and analysis. Although originally designed for genome-wide SNP association studies, they contain signal intensities that can be borrowed to identify regions with deletions or duplications (Komura et al., 2006; Peiffer et al., 2006). Multiple softwares and programs have been developed for these arrays, and their performance is being evaluated (Winchester et al., 2009; Zhang et al., 2011). With a few exceptions, the existing approaches can be roughly classified to two types: single-locus pooled-sample approach and single-individual cross-genome approach. The single-locus pooled-sample approaches use the distributions of signal intensities of multiple samples at a fixed locus to derive reference values and clusters for each CNV value and then determine individual CNV by their belonged cluster, such as TriTyper (Franke et al., 2008) and CNVtools (Barnes et al., 2008). These methods proceed locus by locus and generally ignore inter-marker correlations. The single-individual cross-genome approaches either use partitioning approach [such as, DNAcopy (Olshen et al., 2004), CnvPartition by Illumina, segCNV (Shi and Li, 2012)] or the hidden Markov Models (HMM) [such as, Birdseye (Korn et al., 2008), QuantiSNP (Colella et al., 2007) and PennCNV (Wang et al., 2007)] to call CNV individual by individual. The HMM considers the dependency between copy number states at two adjacent markers by assuming the CNV underlying observed signal intensities is a first-order Markov process (Gelfond et al., 2009). The inter-marker correlation is implied in the homogeneous transition probability that only depends on the inter-marker distance. A noticeable exception to the two lines of methods is a novel Bayesian approach that combines the signal intensity distribution and heterozygosity information to infer individual CNV (Zőllner et al., 2009).

Because both SNPs and CNVs affect the signal intensities and may affect phenotypes separately or jointly, their coexistence will affect the identification of each other and the results of association studies. Ignoring SNPs in CNV analysis fails to incorporate allele-specific gains and losses and diminish the potential to exploit linkage disequilibrium (LD) between CNVs and nearby SNPs. How to combine SNPs and CNVs has been a challenge for geneticists. The common annotations of the copy numbers and SNP genotypes have been independent and the available methods for SNP and CNV calling had been separate until (Korn et al., 2008) presented a sequential approach to generate copy number polymorphisms (CNPs) using results from both genotype calls and CNV calls.

Methods were extended to accommodate the CNP along the same class of CNV calling approaches. In single-locus pooled-sample approach, the CnvPartition reassumed that intensity and proportion of B alleles follow distinct bivariate Gaussian distributions given different CNP. In single-individual cross-genome approach, (Wang et al., 2009) allowed multiple CNP states in their HMM and incorporated the two-locus LD parameter in addition to the inter-marker distance in the transition probability, which increased the accuracy of the CNP calls. cnvHap (Coin et al., 2010) used a new transition probability in their HMM, which only relies on the two-locus haplotype frequencies. The HMM of polyHap (Su et al., 2010) treated a CNV region as a region of variable ploidy and considered only two-locus haplotype.

At presence of CNV, all existing algorithms using haplotypes augmented the conventional haplotype definition by treating duplications and deletion as additional “alleles”, distinct from two existing SNP alleles at each locus. Such defined “haplotypes” are not continuous physical pieces as traditionally perceived, which can cause conceptual confusions. In addition, the number of possible combinations over a region increases exponentially with the base of 4–6 instead of 2, largely increasing computational complexity and leading to infeasibility. There were a few methods to estimate the frequencies of such “haplotypes”. MOCSphaser (Kato et al., 2008b) infers CNV-SNP haplotypes using an expectation-maximization (EM) algorithm but only accommodates integer copy numbers in CNV regions and SNP genotypes in non-CNV regions. CNVphaser employed a hierarchical partition-ligation strategy to break down a longer region into smaller blocks and used the EM algorithm to estimate the “haplotypes” frequencies just as for the regular haplotypes (Kato et al., 2008a).

It often occurs that some intensity values are apparent outliers that can be easily detected by routine quality control procedures. Other methods either exclude the whole samples with some poor quality values (Wang et al., 2007) or re-estimate the SNP at each locus with poor quality by imputation for subsequent calls.

In this paper, we developed a haplotype-based maximum likelihood method to call CNP, which takes account of valuable multi-locus LD information in the population. By posing practical assumptions for short CNV regions, we keep the same conventional haplotypes as originally defined for SNP genotype data and make corresponding inferences. We developed a computationally efficient algorithm that determines optimal CNPs for each individual and estimates haplotype frequencies in the population simultaneously. We consider our method as a natural merge of single-locus pooled-sample and single-individual cross-genome approaches for CNP calling. Our method can also recover CNPs even with missing data. We evaluated our methods through extensive simulations in terms of sensitivity, true positive rate, length of detectable CNV regions for different haplotype structure, frequencies and length of CNV regions. And we compared our method with a few available methods to assess the possible gain of using haplotypes. In addition, we checked how well our method can recover CNPs when there are missing or extreme values in raw data. Last, we applied these methods to the duplicated genotype samples of 23 individuals with acute lung injury (ALI) to check the consistency of our method in calling both CNV and SNP. Accuracy was assessed by comparing calls from all methods to the CNV regions identified through array CGH data.

## 2. Methods

### 2.1. Notations

We use *c* = (*c*_*A*_, *c*_*B*_) to denote the copy numbers of A allele and B allele, or CNP at a bi-allelic marker locus. CNPs at normal states with two copies of alleles include (1,1), (2,0), and (0,2), which code regular SNP genotypes; (0,0) is the CNP at the double deletion state, (0,1) and (1,0) are the CNPs at single deletion states, and (1,2), (2,1), (2,2), (1,3), and (3,1) or more are for the duplication states. The copy numbers in most available methods are referred to as cn = *c*_*A*_ + *c*_*B*_, which does not contain the allele specific information and cannot infer disease risks associated with the copy number change of a specific allele. Because the duplications with four or more copies are virtually indistinguishable on genotype platforms (Wang et al., 2009), we set the maximum copy number to 4.

We denote *X*_*A*_ and *X*_*B*_ as the normalized signal intensity values for allele A and allele B, respectively. *X*_*A*_ and *X*_*B*_ can be extracted from raw CEL files using the standardized normalization procedure, such as the BeadStudio software for Illumina platforms and the Affymetrix Power Tools for Affymetrix platforms. We use two measures log R ratio and B allele frequency, denoted as *r* and *b*, as the observed values in our models. *r* is the logarithm ratio of observed total intensity *R* = *X*_*A*_ + *X*_*B*_ to expected intensity (Peiffer et al., 2006), and *b* is the standardized proportion of samples carrying the B allele, i.e., a linear transformation of θ = arctan(*X*_*B*_/*X*_*A*_)/(π/2).

We assume our study includes *N* individuals and we start with a haplotype block of interest containing *M* markers. Within the haplotype block, there are a total of *s* possible haplotypes *h*_1_, …, *h*_*s*_ with population frequencies ρ = (ρ_1_, …, ρ_*s*_). Throughout the paper, we use subscripts for marker locations and superscripts for individuals.

### 2.2. Haplotype identification given CNP

In this section, we illustrate how individual CNPs can help infer haplotypes under some practical assumptions. We assume either duplication or deletion occurs as a continuous piece on one chromosome and deletion/insertion regions are not immediately next to each other for each individual.

If a CNV region of an individual covers the whole region of interest, we can identify his haplotype(s) as follows:

1. Within a duplication region (cn = cn_1_ = ··· = cn_*M*_ = 3 or 4), the duplicated haplotype *h*_1_ can be written as {*S*_1_ …*S*_*M*_ : *S*_1_ = *A* if *c*_*k*, *A*_ ≥ cn − 1, or *S*_*k*_ = *B* otherwise, for *k* = 1, …, *M*}, and the other haplotype *h*_2_ become {*S*′_1_… *S*′_*M*_ : *S*′_*k*_ = *A* if *c*_*k*, *A*_ = cn or 1, or *S*′_*k*_ = *B* otherwise}.
2. Within a single deletion region (cn = cn_1_ = ··· = cn_*M*_ = 1), the unique haplotype *h*_1_ is {*S*_1_… *S*_*M*_ : *S*_*k*_ = *A* if *c*_*k*, *A*_ > 0, or *S*_*k*_ = *B* if *c*_*k*, *B*_ > 0 for *k* = 1, …, *M*}.

For example, if we know an individual's CNPs (3, 0)(1, 2)(2, 1)(0, 3) at four adjacent loci in a block, it is easy to see that there are three copies of allele A and 0 copy of allele B at the first locus and thus A must be on the duplicated haplotype. Similarly at loci 2, 3, and 4, B, A, and B are on the duplicated haplotype. Therefore the haplotype ABAB must be the duplicated piece and the other AABB is the normal non-duplicated haplotype. Similarly, given an individual's CNPs (1, 0)(0, 1)(0, 1)(1, 0), we will know that one haplotype of his was deleted and the other haplotype is ABBA. For those determined haplotypes given **c** = (*c*_1_,…, *c*_*M*_), we call them “compatible” with **c** and denote as (*h*_1_, *h*_2_) ~ **c**. We say (ABAB, AABB) are compatible with (3, 0)(1, 2)(2, 1)(0, 3), i.e., (ABAB, AABB) ~ (3, 0)(1, 2)(2, 1)(0, 3).

**Figure 1:**
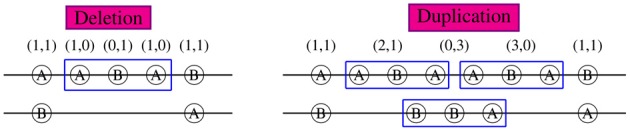
**CNP and haplotype configurations within a 5-locus block.** 2-digit CNP at each locus was shown on top. Specific alleles A or B are shown in each circle at the corresponding loci aligned on a pair of chromosomes. The long lines between two loci denote deletion regions (no corresponding alleles). Both deletion (left) and duplication (right) occur from loci 2 to 4.

If a CNV piece doesn't cover the whole region of interest, the haplotypes compatible with the CNP genotypes are determined by the CNPs within the CNV region and the regular SNP genotypes outside of the CNV region. As shown in Figure 1 for a region including five loci, the haplotype sections within the 3-locus CNV regions are uniquely identified and the sections in the normal region (loci 1 and 5) can be partially inferred given population haplotype distributions. In the left figure with a deleted piece, the middle section of one haplotype is *ABA* and that of the other is deleted following our rule listed previously. Combined with genotypes (1,1) at loci 1 and 5, the possible haplotype couples covering the whole region can be AABAA/B…B, AABAB/B…A, BABAA/A…B, and BABAB/A…A. Similarly in the right figure with a duplication piece, the possible haplotype couples are AABAA/BBBAB, AABAB/BBBAA, BABAA/ABBAB, and BABAB/ABBAA. Now we see that a CNV region of an individual can help infer his/her haplotypes and thus help estimate the population haplotype frequencies. On the contrary, the haplotype information in the population can be used to better infer individual CNPs. In this example, if in the population there is no AABAA, BABAA, or BABAB (i.e., π(AABAA) = π(BABAA) = π(BABAB) = 0), the haplotypes in the left figure can be uniquely determined as AABAB/B…A and in the right figure, the duplicated haplotype would be AABAB and the other BBBAA. In most cases, we know non-zero probabilities of AABAA, AABAB, BABAA, or BABAB in the population and can still make some inference about the haplotypes given CNP. This can be done by incorporating the haplotype distributions in the likelihood as we show next. In our method, we allow CNV regions vary from individual to individual, i.e., the CNV regions of different individuals can have completely different boundaries. Such flexibility would lead to extremely large numbers of haplotypes and likely small probabilities for them in the population in other “haplotype”-based CNV methods.

Please note that, as SNPs, CNPs were aligned according to the reference coordinate, but not to the actual physical locations. For duplications, LRR and BAF can only tell which piece of chromosome is duplicated but not where it is connected to.

### 2.3. Maximum likelihood method using haplotype information

Given observed log R ratio (**r**) and B allele frequency (**b**) at loci 1, …, *M*, the likelihood can be written as a function of CNP and population haplotype frequencies, i.e.,


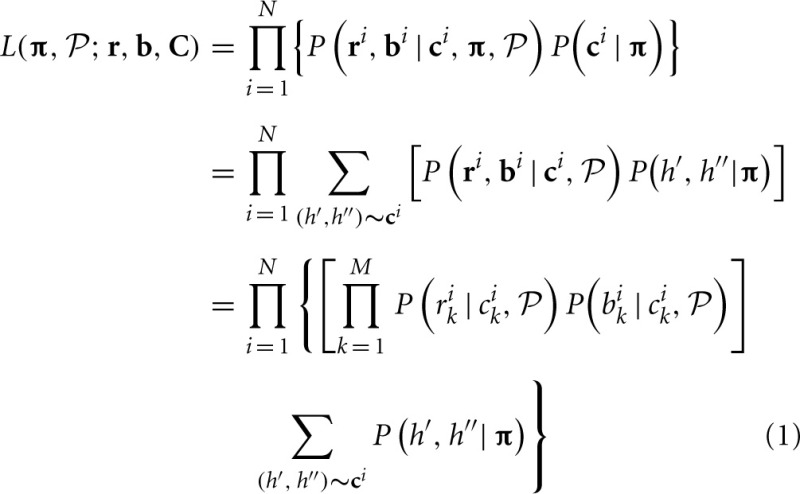


where *i* is the individual index *i* = 1, …, *N*, **r**^*i*^, **b**_*i*_, **c**^*i*^ are LRR, BAF, and CNP at all loci for individual *i* and **C** represents the CNP for all individuals, i.e., **C** = (**c**_1_,…, **c**^*N*^), *k* is the marker index *k* = 1, …, *M*, *P*(*b*^*i*^_*k*_ | *c*^*i*^_*k*_, 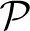) and *P*(*r*^*i*^_*k*_ | *c*^*i*^_*k*_, 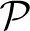) are the conditional probability of the BAF and LRR given the CNP at locus *k*, *P*(*h*′, *h*″) is the probability of observing two haplotypes *h*′ and *h*″, and 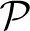 denotes the set of parameters in both conditional probabilities *P*(*b*^*i*^_*k*_ | *c*^*i*^_*k*_, 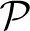) and *P*(*r*^*i*^_*k*_ | *c*^*i*^_*k*_, 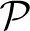). The last equation holds when **b** and **r** are assumed conditionally independent given **c** and either *b*_*k*_ or *r*_*k*_ are conditionally independent of other *b*_*l*_ or *r*_*l*_ given **c**. If we assume Hardy–Weinberg Equilibrium (HWE), *P*(*h*′, *h*″) = 2π_*i*_π_*j*_ if *h*′ = *h*_*i*_ ≠ *h*″ = *h*_*j*_ and *P*(*h*′, *h*″) = π^2^_*i*_ if *h*′ = *h*″ = *h*_*i*_. As in HMM models, we can assume that *P*(*r*_*k*_ | *c*_*k*_ = (*c*_*k*, *A*_, *c*_*k*, *B*_)) ~ *N*(μ_cn(*c*_*k*_)_, σ^2^_cn(*c*_*k*_)_), *P*(*b*_*k*_ | *c*_*k*_) ~ truncated *N*(μ_*b*, cn(*c*_*k*_)_, σ^2^_*b*_), and thus 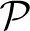 = {μ_0_,…, μ_4_, σ_0_,…, σ_4_, μ_*b*, 0_,…, μ_*b*, 4_, σ_*b*_, η, γ_0, 0_,…, γ_4, 4_, *G*_1, 1_,…, *G*_15, 15_} (details in Appendices B and C).

Maximization of the likelihood Equation (1) with respect to **C** and **π** will yield optimal CNP estimates of each individual and haplotype frequency estimates. But the number of combinations of CNPs of all individual and haplotype is huge and searching over the whole parameter space can be computationally intensive. To reduce the computation burden, we derived an optimization algorithm to update individuals' CNP and haplotype frequencies iteratively.

### 2.4. The CNP optimization algorithm

We used the following iterative algorithm to maximize the likelihood Equation (1):

1. At step 0, assign initial values to CNPs of all individuals **C**^(0)^ and population haplotype frequencies **π**^(0)^ within a haplotype block.
2. At step ℓ, given **C**^(ℓ)^ and **π**^(ℓ)^, maximize the log-likelihood over possible **c**^*i*^ for each individual *i* to obtain optimal **c**^*i*, (ℓ + 1)^, i.e.,
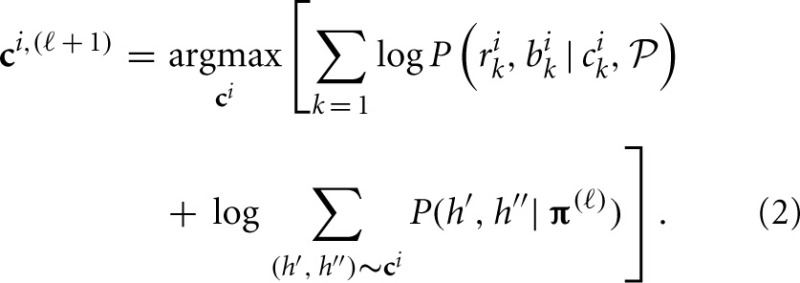
3. Update **π** through collecting the conditional probability of haplotypes given all individuals' CNP. This is a revised expectation-maximization (EM) algorithm (Dempster et al., 1977) of updating haplotype frequencies given individuals' genotypes in the absence of CNV (Excoffier and Slatkin, 1995). Given current estimate of **π**^(ℓ)^ and updated **C**^(ℓ + 1)^, the haplotype frequency is updated according to
  (3) $$\begin{array}{l} {\hat{\pi }}_{t}^{\left(\ell +1\right)}=\frac{1}{2N}\sum_{i=1}^{N}\sum_{(h^{\prime },\;h^{\prime \prime })∼c^{i,\;(\ell +1)}}\{I(h^{\prime }=h_{t})+I(h^{\prime \prime }=h_{t})\}P\left(h^{\prime },\;h^{\prime \prime }|(h^{\prime },\;h^{\prime \prime })∼c^{i,\;(\ell +1)},\;\pi^{\left(\ell \right)}\right)\\ \;=\frac{1}{2N}\sum_{i=1}^{N}\frac{\sum_{(h^{\prime },\;h^{\prime \prime })∼c^{i,\;(\ell \;+\;1)}}\left\{I(h^{\prime }=h_{t}\right)+I(h^{\prime \prime }=h_{t})\}P\left(h^{\prime },\;h^{\prime \prime }|\pi^{\left(\ell \right)}\right)}{\sum_{(h^{\prime },\;h^{\prime \prime })∼c^{i,\;(\ell \;+\;1)}}P\left(h^{\prime },\;h^{\prime \prime }|\pi^{\left(\ell \right)}\right)} \end{array}$$
4. Repeat (2) and (3) until the inferred CNP doesn't change and the estimated parameter $\hat{\pi }$ converges.

When the region is long, the path for convergence can be painfully long and thus makes the computation infeasible. To save the computation burden, we will call the initial CNP using a HMM and then apply our haplotype-based method. The HMM we used are similar to others' (Colella et al., 2007; Wang et al., 2007, 2009) and we refer readers to Appendix A for details.

### 2.5. Missing data

When some *b*_*k*_'s and *r*_*k*_'s are missing, the genotype or haplotype at the missing loci can be inferred using the LD information around the missing loci.

For a single individual, if *b*_*k*_, *r*_*k*_ at loci *k* ∈ D are missing, the contributions of that individual to the overall likelihood is changed to


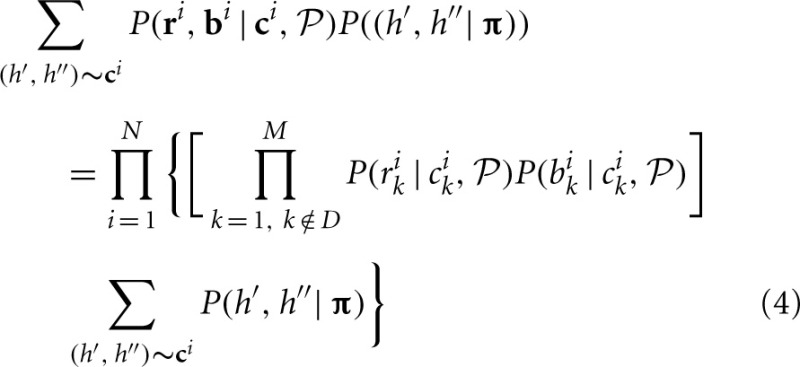


The key difference between likelihoods Equations (1) and (4) is that Equation (1) does not have the components corresponding to possible haplotypes at the unobserved loci for this individual. Computationally, missing data affect initial parameter estimation slightly and may also increase the computational complexity because the number of possible CNPs and haplotypes increases for those individuals with missing data.

If a locus is not genotyped at all, all individuals will not have the corresponding components, but the extended haplotypes in other reference populations can be used to infer the CNP within or outside of the CNV region.

### 2.6. Potential uses of our proposed method

Our proposed method can serve as either an independent calling method or a refining procedure upon the results from other calls. Please note that the initial calls from HMM are not necessary and other initial values can work in our method as well. The haplotype frequency can be estimated as a byproduct in a decent-size sample or can be borrowed from public database such as HapMap to improve the inference in small samples.

## 3. Simulations

To evaluate the performance of our methods, we conducted a series of simulations to access: 1. sensitivity of detecting CNV intervals, i.e., how often we can correctly detect it when there is such a region; 2. true positive rate, i.e., how often our detected CNV region are true; and 3. length of truly and falsely detected CNV regions. The CNV length, frequency among population, and the haplotype structure, were varied to understand their influences on the CNV calls. Our method was compared with five existing methods. We also checked CNP recovery rate using our method under a missing data scenario.

For each data set, we simulated the LRR and BAF for 1000 unrelated individuals following three steps: First, 2000 independent chromosomes of 1000 individuals were generated containing 55,860 SNP loci, the same number of loci on Affymetrix Genome-Wide Human SNP Array 6.0 platform, along chromosome 4. We selected eight haplotype blocks with various length and LD structure, four medium-length and two long blocks with low to medium *R*^2^, two medium-length blocks with high *R*^2^, as shown in Table 1.

**Table 1:** **Summary of the selected haplotype blocks**.

**Gene**	**# of loci**	**Position**	**Average *R*^2^**
ADD1	6	2,841,681–2,893,241	0.1561
CORIN-3	6	47,474,045–47,531,963	0.1477
NR3C2-1	6	149,461,059–149,491,985	0.3305
NR3C2-2	8	149,493,152–149,496,672	0.0852
LOC285501-1	26	179,864,756–179,949,542	0.4225
LOC285501-2	11	180,222,608–180,252,886	0.8002
RP11-404J23.1-1	7	180,322,430–180,354,002	0.7500
RP11-404J23.1-2	24	180,373,119–180,428,267	0.6274

All genotypes were simulated using the allele frequencies in HapMap CEU population. In addition, two-locus LD and multi-locus LD were reserved outside and within the selected haplotype blocks, respectively. Specifically, outside of the haplotype blocks, the alleles at the first locus were generated from Bernoulli(*p*_1_) where *p*_1_ is the population frequency of B allele in CEU; alleles at other loci were generated using the conditional probabilities given the previous alleles as observed in CEU. Within the selected haplotype blocks, the starting SNPs were simulated as before conditional on the alleles at previous locus only but the remaining alleles were simulated as haplotypes based on the conditional haplotype frequencies within the same block in the CEU population.

Second, within the eight selected haplotype blocks, deletion and duplication regions with fixed boundary were randomly chosen from populations and the haplotype piece within the CNV regions was deleted or inserted. At this stage, the true CNPs at all loci were generated.

Third, the LRR and BAF values were generated based on the conditional probability distributions discussed in section A with parameter **μ** = (−2, −0.664, 0, 0.4, 1), **σ** = (0.5714, 0.28, 0.2, 0.21, 0.3333), **μ**_*b*_ = (0.1, 0.04, −0.02, −0.08, −0.14) and σ_*b*_ = 0.1.

We considered multiple scenarios with varying parameters, specified as follows:

1. different length of the CNV regions (3–5 in short blocks and 5–20 in long blocks); We fixed the left boundary of each CNV region so that the LD can remain the same within each block and we can use the LD across different haplotype blocks to understand the influences of the LD between the boundary and other SNPs.
2. more and less frequent CNV; For more frequent CNV regions, we let the population frequencies of deletion and duplication to be 20 and 5%; for less frequent CNV regions, the population frequencies of deletion and duplication were set to be 5 and 1%.
3. random missing of LRR and BAF; we assumed a relatively high missing rate 1% for all loci and all individuals.

For each scenarios, 100 datasets were generated. Based on observed LRR and BAF, we applied our method hap-CNP to call CNPs and compared them with the true CNP. Several CNV calling procedures were compared, including a HMM in Wang et al. (2009) (WHMM), PennCNV for unrelated individuals (Wang et al., 2007), a SCAN method (Jeng et al., 2010), an integrative segmentation method segCNV using both the joint distribution of LRR and BAF (Shi and Li, 2012), and cnvHap (Coin et al., 2010) that is another HMM using two-locus haplotype distribution in transition probabilities.

PennCNV, SCAN, and segCNV only consider the copy numbers and can form good contrasts with ours for CNP. Comparing with WHMM and cnvHap can help us understand how much additional information we can gain using correlation within multi-locus haplotype and flexible boundary assumption.

## 4. Real data analysis

### 4.1. Duplicated samples of patients with acute lung injury

In a genome-wide study to investigate the genetic effect on various trauma-induced clinical outcomes, cases with ALI and controls from at-risk trauma population at the University of Pennsylvania were recruited for genotyping. To control the quality of genotyping, 23 Caucasian ALI patients were randomly chosen to have duplicate serum samples, which were separately genotyped using Illumina HumanQuad610 BeadChip (Illumina, San Diego). Over 600,000 bin-tagging polymorphisms were included and normalized intensity data for each sample were loaded into Illumina Beadstudio 2.0. See Christie et al. (2012) for genotyping details.

To check the feasibility and reliability of our proposed method, we applied it to the 23 pairs of samples and compared our CNP calls with PennCNV, SCAN, and the normal genotypes called using Illumina's clustering algorithm without considering CNV. The summary statistics of raw signal data were checked prior to analysis. Samples with extreme values generally suggest low quality and were removed from the analysis. We used a sliding window of five SNPs through the whole chromosome to determine haplotype blocks. In each window, haplotype frequencies are estimated from the HapMap genotype data. Because the HapMap genotypes were generated mainly from Affymetrix platform, their strands sometimes were the complementary stands of our genotypes. We unified the strandness of two sets of genotypes and estimated the corresponding haplotype frequencies using the EM algorithm implemented in the haplo.stats R package.

We checked the concordance of the genotype of CNV calls from our and others' methods and the recovery of CNP calls among regular genotype calls.

### 4.2. HapMap CEU samples

We also applied PennCNV, SCAN, WHMM, segCNV, and our method in 90 HapMap CEU samples genotyped by Affymetrix Genome-Wide Human SNP Array 6.0. To assess the accuracy of various calling methods, we checked the overlap between CNV calls from these methods and the CNV regions validated through array-CGH (Conrad et al., 2010).

## 5. Results

### 5.1. Simulation results

Table 2 summarizes the results from our method for eight haplotype blocks. The sensitivity ranged from 71.4 to 99.4% for more frequent case and from 72.0 to 99.6% for less frequent case, meaning that we can detect most of CNV regions in all cases. The true positive rate was larger than 97% in general, meaning that vast majority of our detected regions are true CNV regions.

**Table 2:** **Summary of CNP regions and genotypes called from hap-CNP**.

**CNV type**	**Haplotype block**	**True CNV length**	**Sensitivity (s.d.)**	**True positive rate (s.d.)**	**Within truly detected regions**	**Within falsely detected regions**
					**Length of CNV regions**	**Length of CNV regions**
More frequent	CORIN-3	3	0.714(0.026)	0.999(0.002)	3.064(0.662)	1.750(0.886)
		4	0.810(0.022)	0.999(0.002)	3.946(0.683)	2.500(1.509)
		5	0.839(0.025)	0.999(0.002)	4.794(0.711)	2.571(1.272)
	ADD1	3	0.715(0.028)	1.000(0.001)	3.027(0.636)	1.000(0.000)
		4	0.817(0.026)	0.999(0.002)	3.900(0.660)	2.250(1.488)
		5	0.865(0.020)	1.000(0.001)	4.782(0.673)	1.000(NA)
	NR3C2-1	3	0.725(0.028)	0.973(0.015)	3.187(0.791)	1.368(0.797)
		4	0.824(0.025)	0.972(0.011)	3.961(0.663)	1.384(0.772)
		5	0.880(0.019)	0.972(0.013)	4.783(0.681)	1.467(0.979)
	NR3C2-2	3	0.713(0.027)	0.993(0.006)	3.148(0.675)	1.847(1.201)
		4	0.824(0.029)	0.984(0.010)	4.034(0.707)	1.633(1.105)
		5	0.878(0.020)	0.941(0.017)	5.014(0.806)	1.385(0.920)
	LOC285501-2	3	0.703(0.030)	1.000(0.001)	3.067(0.790)	2.000(0.000)
		4	0.767(0.029)	0.999(0.002)	4.230(1.149)	1.833(0.983)
		5	0.854(0.023)	0.999(0.002)	5.015(0.905)	2.800(1.398)
	RP11-404J23.1-1	3	0.691(0.027)	0.999(0.002)	3.072(0.707)	2.100(1.449)
		4	0.792(0.027)	0.999(0.002)	3.978(0.761)	1.750(1.165)
		5	0.836(0.029)	0.999(0.001)	4.895(0.770)	2.800(0.837)
	LOC285501-1	5	0.866(0.022)	0.998(0.003)	5.041(0.915)	2.647(1.730)
		20	0.995(0.005)	0.998(0.002)	19.875(1.794)	2.158(1.344)
	RP11-404J23.1-2	15	0.988(0.007)	0.999(0.002)	14.908(1.628)	2.769(1.536)
		20	0.994(0.005)	0.999(0.002)	19.863(1.783)	2.125(1.586)
Less frequent	CORIN-3	3	0.720(0.059)	0.996(0.009)	3.102(0.698)	1.333(0.500)
		4	0.826(0.044)	0.997(0.007)	3.965(0.724)	2.429(1.397)
		5	0.852(0.049)	0.997(0.007)	4.796(0.759)	1.667(0.516)
	ADD1	3	0.726(0.053)	0.997(0.008)	3.055(0.698)	1.000(0.000)
		4	0.829(0.043)	0.998(0.006)	3.980(0.706)	1.833(1.169)
		5	0.892(0.040)	0.998(0.005)	4.806(0.674)	1.800(0.837)
	NR3C2-1	3	0.736(0.052)	0.957(0.033)	3.074(0.749)	1.371(0.711)
		4	0.840(0.047)	0.952(0.032)	3.954(0.697)	1.370(0.699)
		5	0.881(0.047)	0.961(0.022)	4.811(0.699)	1.413(0.925)
	NR3C2-2	3	0.736(0.059)	0.987(0.018)	3.179(0.715)	1.893(1.474)
		4	0.835(0.054)	0.976(0.018)	4.085(0.763)	1.525(1.058)
		5	0.882(0.041)	0.912(0.040)	5.033(0.836)	1.469(1.044)
	LOC285501-2	3	0.695(0.064)	0.996(0.010)	3.063(0.841)	2.000(1.225)
		4	0.776(0.050)	0.995(0.012)	4.248(1.174)	2.182(1.168)
		5	0.877(0.043)	0.998(0.006)	5.090(1.002)	1.600(0.548)
	RP11-404J23.1-1	3	0.702(0.065)	0.996(0.009)	3.084(0.764)	1.857(1.464)
		4	0.810(0.060)	0.998(0.006)	4.028(0.841)	3.000(0.707)
		5	0.864(0.039)	0.997(0.008)	4.933(0.835)	2.333(1.225)
	LOC285501-1	5	0.898(0.040)	0.992(0.012)	5.154(1.004)	2.476(1.436)
		20	0.997(0.007)	0.992(0.013)	20.002(1.689)	2.385(1.329)
	RP11-404J23.1-2	15	0.989(0.012)	0.995(0.008)	15.009(1.731)	2.846(1.405)
		20	0.996(0.008)	0.994(0.010)	19.951(1.692)	2.667(1.589)

As the length of true CNV interval increased with the same haplotype block, the sensitivity increased and the true positive rate remained similar. With comparable LD, the longer CNV regions across different blocks tend to have higher sensitivity and true positive rates. This is because haplotypes are more likely to be separated with longer CNV regions and in return the more accurate haplotype information can lead to better CNV detection.

In addition, we found the majority of the false negatives resided on the boundary rather than within the true region, which was also consistently observed in the results from other methods.

The sensitivity remained stable in both common and rare CNV intervals, while the true positive rate was slightly higher for common CNV regions than that for rare CNV regions. Though we expected better performance in common CNV than rare CNV, the sample size we generated was large enough to give reliable haplotype inference. So the frequency of CNV doesn't play much role in studies with a decent sample size (*n* ~ 1000). For smaller sample size (*n* < 200), common CNVs could lead to much better inference in Haplotypes than rare CNVs and the difference can be larger.

There was no clear trend of sensitivity change as LD gets stronger from CORIN-3, ADD1, NR3C2-1 to LOC285501-2 (overall average *R*^2^ increases from 0.15 to 0.81). Even the LD is assumed to help the inference of CNP, the excess high LD may not necessarily lead to much accuracy gain and the little gain may be covered by the boundary LD and length of the CNV regions.

In truly detected CNV regions, the average length of the regions was close to the truth; while in falsely detected CNV regions, the average length was around 1, meaning the most of the falsely detected CNV were singletons. On the contrary, almost all detected singletons were false CNVs and the longer detected regions were more likely to be the true CNV regions. The average lengths of detected CNV intervals were similar for common and rare cases while the variability was bigger for rare case due to the small sample size of CNV intervals.

As a comparison, the results from other methods including PennCNV, SCAN, WHMM, cnvHap, and segCNV are summarized in Table 3. In general, PennCNV underestimated CNV regions and more often it happened when the true CNV regions were short. WHMM, SCAN and segCNV methods were more sensitive than PennCNV, but slightly less than our method. Interestingly, although PennCNV detected fewer CNV loci and regions, their detected CNV regions were often longer than the truth. WHMM yielded much more CNV regions than the truth, which resulted in high sensitivity with small true positive rate. SCAN had similar performance as ours for less frequent cases, while it has smaller sensitivity than ours for more frequent cases. SCAN showed slightly longer falsely detected CNV regions than our methods in both cases. segCNV, as a partitioning method, performs similar to SCAN. cnvHap, as the only method using haplotypes we are comparing with, has superior true positive rates than others in general. For short CNV regions, it has better sensitivity than others but this advantage quickly diminishes as CNV regions become longer. On one hand, cnvHap's results support the advantage of using haplotype information but its usage of two-locus haplotypes may not be ideal for a long block, which seems improved in our method. It is counter-intuitive to see that cnvHap perform worse for longer CNVs. This is partially because the results are sensitive to the choice of blocks, even with true values, in the customized normalization step of cnvHap.

**Table 3:** **Summary of CNV region and genotype calls from all methods**.

**Haplotype block**	**Method**	**True CNV length**	**Sensitivity (s.d.)**	**True positive rate (s.d.)**	**Within truly detected regions**	**Within falsely detected regions**
					**Length of CNV regions**	**Length of CNV regions**
NR3C2-1 More frequent	hap-CNP	3	0.725(0.029)	0.973(0.015)	3.187(0.791)	1.368(0.797)
		4	0.824(0.025)	0.972(0.011)	3.961(0.663)	1.384(0.772)
		5	0.880(0.019)	0.972(0.013)	4.783(0.681)	1.467(0.979)
	PennCNV	3	0.167(0.024)	0.868(0.052)	4.252(1.017)	3.642(1.737)
		4	0.534(0.035)	0.912(0.023)	4.395(0.611)	3.162(1.749)
		5	0.755(0.024)	0.919(0.023)	4.952(0.447)	2.970(1.728)
	SCAN	3	0.556(0.033)	0.989(0.008)	3.265(1.036)	2.065(1.576)
		4	0.723(0.030)	0.986(0.009)	4.062(0.847)	1.851(1.422)
		5	0.821(0.024)	0.984(0.009)	4.785(0.712)	1.910(1.379)
	WHMM	3	0.637(0.027)	0.968(0.016)	1.846(0.839)	2.069(1.317)
		4	0.747(0.026)	0.956(0.013)	2.497(1.132)	2.059(1.361)
		5	0.806(0.025)	0.947(0.015)	3.317(1.375)	2.209(1.419)
	cnvHap	3	0.888(0.114)	1.000(0.001)	3.010(0.114)	1.000(NA)
		4	0.572(0.065)	1.000(0.002)	4.067(0.250)	5.000(NA)
		5	0.217(0.079)	1.000(0.000)	4.992(0.091)	NA(NA)
	segCNV	3	0.275(0.024)	0.984(0.017)	3.239(0.633)	2.546(1.792)
		4	0.603(0.029)	0.989(0.009)	3.360(0.697)	2.152(1.564)
		5	0.820(0.022)	0.987(0.006)	4.064(0.724)	2.500(1.789)
NR3C2-1 Less frequent	hap-CNP	3	0.736(0.052)	0.957(0.033)	3.074(0.749)	1.371(0.711)
		4	0.840(0.047)	0.952(0.032)	3.954(0.697)	1.370(0.699)
		5	0.881(0.047)	0.961(0.022)	4.811(0.699)	1.413(0.925)
	PennCNV	3	0.123(0.043)	0.560(0.145)	3.841(0.962)	3.556(1.699)
		4	0.499(0.068)	0.806(0.078)	4.231(0.515)	3.525(1.814)
		5	0.735(0.058)	0.834(0.053)	4.956(0.406)	3.216(1.760)
	SCAN	3	0.576(0.062)	0.982(0.021)	2.972(0.788)	2.121(1.763)
		4	0.751(0.047)	0.980(0.020)	3.905(0.765)	1.826(1.465)
		5	0.838(0.055)	0.983(0.016)	4.749(0.739)	2.023(1.640)
	WHMM	3	0.294(0.064)	0.971(0.038)	1.615(0.727)	1.692(0.970)
		4	0.434(0.062)	0.970(0.034)	2.075(0.989)	1.452(0.832)
		5	0.535(0.061)	0.970(0.028)	2.506(1.236)	1.694(1.103)
	cnvHap	3	0.782(0.064)	1.000(0.000)	3.224(0.432)	NA(NA)
		4	0.824(0.057)	1.000(0.000)	4.474(0.500)	NA(NA)
		5	0.811(0.075)	0.998(0.005)	4.826(0.379)	5.000(0.000)
	segCNV	3	0.348(0.060)	0.987(0.021)	3.118(0.482)	3.500(1.871)
		4	0.672(0.066)	0.990(0.015)	3.283(0.540)	2.750(1.669)
		5	0.853(0.047)	0.990(0.015)	4.043(0.695)	4.100(2.183)

Tables 2, 3 demonstrate that our method provides the most sensitive and accurate results among the methods considered.

When we randomly selected 1% loci to have missing *b* and *r* values, the CNP at missing loci were estimated using neighbor markers as described in section 2.5. As described in Table 4, recovery rate is generally higher for longer CNV regions. Missing loci in rare case are more likely to be correctly recovered than those in more frequent cases. For less frequent cases, there are more normal copy number loci which can be used for accurate haplotype frequency estimation.

**Table 4:** **Missing recovery using haplotypes**.

**CNV type**	**Haplotype block**	**True CNV length**	**No of missing**	**No of recovery**	**No of correct**	**Correctness rate**
More frequent	CORIN-3	3	158	158	79	0.500
		4	196	196	155	0.791
		5	235	235	181	0.770
		6	261	261	211	0.808
	ADD1	3	151	151	99	0.656
		4	175	175	114	0.651
		5	263	263	195	0.741
	NR3C2-1	3	129	129	65	0.504
		4	204	204	134	0.657
		5	252	252	187	0.742
	NR3C2-2	3	140	140	81	0.579
		4	198	198	135	0.682
		5	248	248	188	0.758
Less frequent	CORIN-3	3	34	34	20	0.588
		4	44	44	39	0.886
		5	56	56	47	0.839
		6	71	71	58	0.817
	ADD1	3	29	29	16	0.552
		4	41	41	28	0.683
		5	65	65	47	0.723
	NR3C2-1	3	32	32	21	0.656
		4	43	43	27	0.628
		5	55	55	44	0.800
	NR3C2-2	3	43	43	25	0.581
		4	58	58	35	0.603
		5	63	63	52	0.825

For haplotype inference, we checked the haplotype frequency estimates from our method and from HaploView using all individuals with normal copies. Within the NR3C2 block, the sum of squared errors of estimated frequencies vs. the true frequencies from our method and from HaploView had a mean of 0.0007 and 0.0011, respectively. This shows a better accuracy of inferring haplotypes using CNP, as a byproduct of our method. We found that our method can give similar results no matter how long the CNV interval is but the estimates can be more accurate in common CNV regions than less frequent CNV regions (data not shown). That's not surprising because with common CNV regions, more individuals have CNV and those more informative haplotypes.

Based on 500,000 simulations, the average computing times for one chromosome were 10.5 s for PennCNV, 77.7 s for WHMM, 1.0 s for SCAN, 3.2 s for segCNV, and 81.5 s for our hap-CNP. cnvHap requires customized input for each haplotype block and thus we only ran it over our tested blocks, which took 137.7 s per simulation. As expected, SCAN is extremely fast; PennCNV is efficient using the forward-backward algorithm for HMM and it was developed for calling CNV only without allelic specifications. WHMM and our hap-CNP are comparable though we allow copy numbers to range from 0 to 4, more states than 1–3 in WHMM. cnvHap requires more computing time and would become challenging to run over a whole chromosome, mostly because the number of “haplotypes” increases considerably with all possible CNVs.

### 5.2. CNPs on chromosomes 1 of duplicated samples

Without loss of generality, we reported the results of CNP calls on chromosome 1. Among 46 samples, two had either extremely large variance or median absolute deviation and thus were removed from further analysis. There are 1317 loci missing in LRR or BAF among all subjects. Among them 22 loci were recovered from our algorithm.

Table 5 summarizes the total number of CNV calls on chromosome 1 from one set of samples in contrast of the calls from the other set of samples, using our method, PennCNV, and SCAN.

**Table 5:** **Concordance of copy numbers between duplicated samples**.

	**hap-CNP**					**SCAN**					**PennCNV**				
	**0**	**1**	**2**	**3**	NC^*^		**0**	**1**	**2**	**3**	NC^*^		**0**	**1**	**2**	**3**	NC^*^
0	95	10	7	0	2	0	0	0	0	0	3	0	0	0	0
1	10	649	1756	6	7	0	868	1718	7	5	0	142	78	0	0
2	23	8096	973,114	1195	1050	0	9734	962,981	4093	1054	0	217	987,122	123	970
3	1	149	2533	379	2	0	106	7922	575	7	0	0	154	265	1
NC^*^	0	0	133	0	53	0	1	143	1	53	0	0	146	0	47

* *No call*.

The copy number concordance rates were 98.6, 97.5, and 99.8% for our method, SCAN and PennCNV, respectively. PennCNV showed higher concordance rate due to its conservative detection of CNV while our method and SCAN detected more CNV loci. The copy number discordant rate was 1.00% in our method, mainly caused by three individuals whose *r* is further away from majority of individuals.

Table 6 summarizes the number of normal SNP genotype calls from one set of samples compared with the other set of samples. The concordance rate of regular genotypes (cn = 2) using our method was 99.95% and for BeadStudio was 99.99%. But our method had a no-call rate of 0.13%, much smaller than 4.35% from BeadStudio. BeadStudio provides high amount of no-calls to maintain the concordance rate almost perfect. While our method extracts more information from the BeadStudio's no-calls, that is, among 43,018 no-call loci from BeadStudio, 38,962 loci were genotyped concordantly by our method. Hence, there was a trade-off between no call rate and concordance rate.

**Table 6:** **Concordance of normal genotypes between duplicated samples**.

	**hap-CNP**				**BeadStudio**			
		**AA**	**AB**	**BB**	NC^*^		**AA**	**AB**	**BB**	NC^*^
AA		315,664	89	7	394		300,050	12	7	698
AB		162	301,801	123	297		11	299,085	36	579
BB		1	127	358,748	354		6	24	347,019	565
NC^*^		37	36	51	53		94	167	272	40,643
Total		315,864	302,057	358,748	1098		300,161	299,298	347,334	42,485

* *No call*.

### 5.3. CNPs on chromosomes 1 of CEU samples

**Figure 2:**
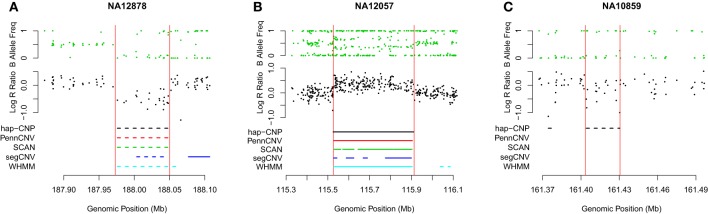
**Detected CNV intervals.** Three regions from three different HapMap samples were taken to show the detected regions. **(A,B)** Show deleted and duplicated regions detected by all five methods. **(C)** Shows a deleted region only detected by our method. All regions are located in chromosome 1.

Table 7 summarizes CNV regions on chromosome 1 of CEU samples detected by PennCNV, SCAN, segCNV, WHMM, and our hap-CNP in overlap with the CNV regions validated through array CGH and with each other. Among a total of 26.5 Mb CNV regions detected by hap-CNV, more than 80% are in the validated regions. PennCNV and SCAN detected much less but most of their detected regions are covered by the validation set. Overall, we have detected 70, 57, 67, and 78% unique CNVs which cannot be found in PennCNV, SCAN, segCNV, and WHMM. Despite that the majority of CNV regions were among validated regions, the percentage of all validated regions covered by each approach was tiny (0.13, 0.06, 0.10, 0.09, and 5.16%, respectively), suggesting genotyping platform may have limited sensitivity for CNV detecting compared with aCGH. When we checked individual calls, most of long CNV regions were called by all five algorithms but there is no persistent optimal choice of an algorithm. As example regions from three samples are shown in Figure 2, long deletion and duplication regions were detected by all five methods in **(A)** and **(B)** but a small deletion CNV region was detected only by our method.

**Table 7:** **Number of CNV calls on chromosome 1 of CEU samples**.

	**Total CNV (Mb)**	**Overlap with**					
		**aCGH (%)**	**hap-CNP (%)**	**PennCNV (%)**	**SCAN (%)**	**segCNV (%)**	**WHMM (%)**
hap-CNP	26.53	82.6	–	30.0	43.3	32.7	21.7
PennCNV	8.39	90.2	87.1	–	82.9	71.2	27.8
SCAN	18.37	87.5	61.8	38.8	–	48.5	20.0
segCNV	17.37	82.7	49.4	37.0	51.5	–	16.8
WHMM	1097.15	72.7	0.4	0.2	0.3	0.3	–

Computation time was in similar scales as in simulations. For 90 CEU individuals, the average computing time on chromosome 1 were 1.0, 16.9, 3.2, 48.2, and 28.8 s for SCAN, PennCNV, segCNV, WHMM, and our method, respectively.

## 6. Discussion

SNPs and CNVs may affect phenotypes separately or jointly and the accuracy of their call can affect the results of association studies. Ignoring CNVs during SNP genotyping may lead to failure to capture the true underlying sequence at many sites and can create the appearance of violations of Mendelian inheritance or Hardy–Weinberg equilibrium where in fact none exists. Using only CNV while ignoring the allelic information in the association studies may fail to incorporate allele-specific gains and losses and diminish the potential to exploit LD between CNVs and nearby SNPs (ongoing study). Association analysis using copy number only without differentiating alleles can dilute the effect size and the power, as shown in both simulations and real studies of insulin and schizophrenia (Hu et al., under review; Irvin et al., 2011).

We didn't separate LOH from the CNV calls, but it can be checked as a special class from our call, i.e., regions with *c*_*A*_ · *c*_*B*_ = 0 and *c*_*A*_ + *c*_*B*_ = 2. Due to the limitation of genotyping platforms, our method can not detect interchromosome duplication and dispersed segmental duplication, which can be discovered using genomic sequence data.

Our assumption for haplotype-based CNV inference means that within a region, the deletion/duplication piece cannot end at locus *T* on one chromosome and then occur immediately again from *T* + 1 on the other. If in reality this occurs, one more parameter of the event probability can be incorporated in the likelihood, which will result in much longer computation time as a trade-off.

In duplication regions, our method also relies on the assumption of a nearby haplotype being duplicated. In reality, exceptions could occur, which may affect the performance or our method in uncertain ways. So users should be cautious about the inference on the duplication regions when the assumption is in doubt. Further investigations on how likely this would happen and what bias it leads to are warranted.

The genotypes at the loci with missing LRR/BAF values can be inferred using the neighborhood haplotype information. Depending on whether the loci are at the boundary of or within CNV regions, the copy numbers may not be accurately recovered. This can also be used for imputing CNP genotypes of some individuals that were genotyped using a platform different from others.

We used the estimates from an HMM as the initial values for our proposed haplotype-based method to expedite the computation. We also checked the robustness of our method using other initial estimate such as clusters based on arbitrary cut-off values for LRR and BAF. We found the performance of our method was consistent. For the long regions, the initial calls from HMM were generally reliable and had little space for improvement. But for short regions where our model assumptions are more likely to meet, our method yielded more reliable and accurate calls. These finding were consistent as reported in Wang et al. (2009).

Whether real chromosomes can be partitioned as unrelated haplotype blocks is still a question, early studies (Daly et al., 2001; Patil et al, 2001; Dawson et al, 2002; Gabriel et al, 2002; Zhang et al., 2003) has shown the rational and feasibilities of separated blocks' representation. So we adopted known haplotype blocks in our simulation. As a limitation of the algorithm, the data generated can only have similar local LD patterns as in the HapMap CEU population.

In analysis of HapMap data, we used sliding-window approach to avoid the selection of haplotype blocks. We have tested a few similar window sizes, which resulted little differences. But longer blocks can cause problem in haplotype estimates and slow down the algorithms even though they worked fine in our simulations. In addition, since there is more chances to detect longer CNV and less space for improvement, using long sliding windows may be not efficient in whole-genome scan.

The mutual benefits of haplotype and CNP inference can be applied to other data such as next generation sequence data, as in our ongoing work.

### Conflict of interest statement

The authors declare that the research was conducted in the absence of any commercial or financial relationships that could be construed as a potential conflict of interest.

## Appendix

### A. Conditional probability for *R* and *B*

Given each CNP, we will assume the conditional probability *P*(*r*_*k*_ | *c*_*k*_ = (*c*_*k*, *A*_, *c*_*k*, *B*_)) to be Gaussian with mean μ_cn_*k*__ and variance σ^2^_cn_*k*__ where cn_*k*_ is the copy number at the locus *k* given by cn_*k*_ = cn(*c*_*k*_) = *c*_*k*, *A*_ + *c*_*k*, *B*_, i.e.,

$$P\left(r_{k}|c_{k}=\left(c_{k,\;A},\;c_{k,\;B}\right)\right)\sim N\left(\mu_{{\text{CN}}_{k}},\;\sigma_{{\text{CN}}_{k}}^{2}\right).$$

Due to the truncation and linear transformation of θ used for the *b* calculation, we will model the conditional probability of *b* as a mixture distribution with two point mass at 0 and 1 (denoted as δ_0_ and δ_1_, respectively) and truncated normal distribution. The truncation only affects for the copy numbers having one component is 0. Also mostly *b* follows normal distribution, e.g., *b*_*k*_ | *c*_*k*_ ~ *N*(μ_*b*, cn_*k*__, σ^2^_*b*_) when *c*_*k*, *B*_ = 0 in the sense that *P*(*b*_*k*_ = 0 | *c*_*k*_) = *P*(*N*(μ_*b*, cn_*k*__, σ^2^_*b*_) ≤ 0), *P*(*b*_*k*_ = 1 | *c*_*k*_) = *P*(*N*(μ_*b*, cn_*k*__, σ^2^_*b*_) ≥ 1), and *P*(*b*_*k*_ ≤ *b* | *c*_*k*_) = *P*(*N*(μ_*b*, cn_*k*__, σ^2^_*b*_) ≤ b) for *b* ∈ (0, 1). When *c*_*k*, *A*_ = 0 and *c*_*k*, *B*_ = c, the distribution of *b*_*k*_ | *c*_*k*_ is the same as 1 − *b*_*k*_ | *c*_*k*_ = (*c*, 0) if *c* > 0 and *b*_*k*_, | *c*_*k*_ ~ *N*(1/2, σ^2^_*b*_) if *c* = 0. If *c*_*k*, *A*_, *c*_*k*, *B*_ > 0, then *b*_*k*_ | *c*_*k*_ ~ *N*(*c*_*k*, *B*_/cn_*k*_, σ^2^_*b*_).

These conditional probabilities can be assumed the same as the emission probabilities in an HMM model and thus the parameters can be estimated nicely from the HMM model.

### B. Hidden markov model

A first order HMM on the loci specific CNP is used to infer the initial **c**^*i*^ for each individual. In this model, the hidden CNP at each locus depends only on the CNP at the most preceding locus. Given the observed data **r**^*i*^, **b**^*i*^, the most likely hidden CNP at each marker locus becomes the initial value, i.e., the CNP that maximizes the likelihood given in Equation (5). In this computation, the Viterbi's algorithm is used, see Viterbi (1967).

Also by assuming that the values of *r* and *b* are independent given the hidden copy number state, the likelihood of the observed data becomes

(5) $$\begin{array}{l} \text{​​​​​​​​}P\text{​}(r,\;b)=\sum_{c}P(r,\;b|c)P(c)\\ \;=\sum_{c}\left\{\prod_{k=1}^{M}P\left(r_{k}|c_{k}\right)P\left(b_{k}|c_{k}\right)\right\}\left\{P\left(c_{1}\right)\prod_{k=2}^{M}P\left(c_{k}|c_{k-1}\right)\right\}. \end{array}$$

Note that the HMM (Equation 5) is different from the haplotype model (Equation 1). As we can see from Equation (5), there are two main components in the HMM calculation. The emission probabilities *P*(*r*_*k*_ | *c*_*k*_) and *P*(*b*_*k*_ | *c*_*k*_) remain the same in Appendix A, and transition probability is given by *P*(*c*_*k*_ | *c*_*k* − 1_) = *t*(cn_*k* − 1_, cn_*k*_, *d*_*k*_) *G*(*c*_*k* − 1_, *c*_*k*_) where *t*(*i*, *j*, *d*) is a distance based transition matrix among copy numbers given by 1 − (1 − γ_*i j*_)(1 − *e*^−η*d*^) if *i* = *j* and γ_*ij*_ (1 − *e*^−η*d*^) otherwise. and *G*(·,·) is a transition matrix within the states having the same copy number. The fact that the coefficient matrix Γ = (γ_*ij*_) is a transition probability matrix, that is, each elements are non-negative and row sums are 1, is used in the parameter estimation.

### C. Parameter estimation

The initial model parameters for the HMM were determined from our preliminary results based on the HapMap data. In theory, the maximum likelihood estimators (MLE) of the parameters,  = (**μ**, **σ**, **μ**_*b*_, **σ**_*b*_, **η**, **γ**), can be obtained by maximizing the following likelihood function

In practice, maximizing Equation (6) in terms of multiple parameters is computationally intensive for a huge number of summations will be involved for a large *M*. As in a typical HMM, we used a Baum–Welch algorithm (Baum et al., 1970) to make the computation feasible. The details are in the following sections.

#### C.1. The baum–welch algorithm

At each iteration ℓ with current parameter estimate , calculate the conditional expectation of the log likelihood, given by

where  is the set of all possible CNP states.

Two required conditional probabilities ρ_*k*_(*s*) = *P*(*c*_*k*_ = *s* | **b**, **r**, ) and ξ_*k*_(*s*, *t*) = *P*(*c*_*k* − 1_ = *s*, *c*_*k*_ = *t* | **b**, **r**, ) are computed using the forward and backward algorithms in the general HMM theory.

In the M-step, parameters are updated by maximizing the conditional expectation (Equation 7), which included three separate terms that can be maximized separately: the first term only concerns the parameters in the transition matrix, the second and the third terms concern the parameters in the emission probability of the B allele frequency and the log R ratio, respectively. Here, we give the formulas for updating the parameter with derivation details in following sections.

#### C.2. Parameters in the emission probability of LRR

Without any constraint on μ and σ, the maximizers ${\tilde{\mu }}_{j}$ and ${\tilde{\sigma }}_{j}$ are given by

$$\begin{array}{l} \;{\tilde{\mu }}_{j}=\sum r_{k}{\tilde{\rho }}_{k}(j)/\sum {\tilde{\rho }}_{k}(j)\text{ and}\\ {\tilde{\sigma }}_{j}^{2}=\sum {\left(r_{k}-\mu_{j}^{\ell +1}\right)}^{2}{\tilde{\rho }}_{k}(j)/\sum {\tilde{\rho }}_{k}(j) \end{array}$$

for *j* = 0, 1, 2, 3, and 4, where .

We assumed a constraint μ_1_ ≤ μ_2_ = 0 ≤ μ_3_, then

$$\begin{array}{l} \mu_{1}^{\ell +1}=\min(0,\;{\tilde{\mu }}_{1}),\;\mu_{2}^{\ell +1}=0,\;\mu_{3}^{\ell +1}=\max(0,\;{\tilde{\mu }}_{3})\text{ and}\\ \;{\left(\sigma_{j}^{\ell +1}\right)}^{2}={\tilde{\sigma }}_{j}^{2}+{\left(\mu_{j}^{\ell +1}-{\tilde{\mu }}_{j}\right)}^{2}. \end{array}$$

If a signal-to-noise constraint is assumed, that is, μ_1_ + *v*σ_1_ ≤ μ_2_ − *v*σ_2_ and μ_2_ + *v*σ_2_ ≤ μ_3_ − *v*σ_3_, then no closed form is available. If $\tilde{\mu }$ and $\tilde{\sigma }$ satisfies the condition ${\tilde{\mu }}_{1}+v{\tilde{\sigma }}_{1}<{\tilde{\mu }}_{2}-v{\tilde{\sigma }}_{2}$, then new estimators are $\mu_{1}={\tilde{\mu }}_{1}$ and $\sigma_{1}={\tilde{\sigma }}_{1}$. Otherwise, new μ_1_, μ_2_, σ_1_, σ_2_ are on the boundary μ_1_ + *v*σ_1_ = μ_2_ − *v*σ_2_. The maximizer under this condition can be found an iterative conditional maximization. For example, if σ_1_ and σ_2_ are fixed, the argument function for maximization becomes

$$w_{1}\frac{{\left(\mu_{1}-{\tilde{\mu }}_{1}\right)}^{2}}{\sigma_{1}^{2}}+w_{2}\frac{{\left(\mu_{2}-{\tilde{\mu }}_{2}\right)}^{2}}{\sigma_{2}^{2}}=\text{const}+\mu_{1}^{2}\left(\frac{w_{1}}{\sigma_{1}^{2}}+\frac{w_{2}}{\sigma_{2}^{2}}\right)-2\mu_{1}\left(\frac{w_{1}{\tilde{\mu }}_{1}}{\sigma_{1}^{2}}+\frac{w_{2}({\tilde{\mu }}_{2}-v(\sigma_{1}+\sigma_{2}))}{\sigma_{2}^{2}}\right)$$

where $w_{j}=\sum_{k=1}^{M}{\tilde{\rho }}_{k}(j)$. Hence, μ_1_ and μ_2_ are updated by minimizing the argument function which is a quadratic equation, so it has a closed form solution. One possible conditional maximization sequence would be a sequential updates of (μ_1_, μ_2_), (μ_2_, σ_2_), (μ_1_, μ_2_), and (μ_1_, σ_1_) under the other parameters are held fixed. Then the unconditional maximizers are obtained by repeating this procedure until convergence is achieved.

Furthermore, we assume μ_0_, μ_4_, σ_0_, σ_4_ are fixed in practice due to the sparsity of the deletion and duplication states. In order to obtain a stable estimation for the standard deviation parameters, we assume that σ_*j*_ for *j* = 1, 2, 3 are bounded below and above by σ_lower_ and σ_upper_ which are the half and double of the standard deviation of **r**.

#### C.3. Parameters in the emission probability of BAF

Since BAF has bounded values on [0, 1], estimations of related parameters are a bit complicated. For instance, the argument function for estimation of μ_*b*, *j*_ is given by

(8) $$\sum_{k=1}^{M}[\left\{\rho_{k}(j,\;0)I(b_{k}=0)+\rho_{k}(0,\;j)I(b_{k}=1)\}\log\Phi (-\frac{\mu_{b,j}}{\sigma_{b}}\right)-\frac{I(0<b_{k}<1)}{2\sigma_{b}^{2}}\{\rho_{k}(j,\;0){\left(b_{k}-\mu_{b,j}\right)}^{2}+\rho_{k}(0,\;j){\left(b_{k}-1+\mu_{b,j}\right)}^{2}\}]$$

where Φ is the cumulative distribution function of the standard normal distribution. Since there is no analytic solution of the maximizer of Equation (8), a first order Taylor expansion around the current value is used to obtain a local maximizer.

#### C.4. Parameters in the transition matrix

The maximizer of *G* is obtained by

However, the convergence of this maximizer is very slow. A column-wise or block-wise estimation which assume all elements in the same column or block are the same is more reliable than element-wise estimation given Equation (9).

For the parameters η and γ_*ij*_ in the transition matrix, we use an iterative minorization-maximization (MM) algorithm [see Hunter and Lange (2004) and references therein]. Using a Young's inequality, a minorized argument function for γ is obtained by

(10) $$\sum_{k=2}^{M}\left[\sum_{i,\;j}\kappa_{k}(i,\;j)\log\gamma_{ij}-\sum_{i=0}^{4}{\tilde{\zeta }}_{k,\;i}\kappa_{k}(i,\;i)\log\gamma_{ii}\right]$$

which has a closed for maximizer where ${\tilde{\zeta }}_{k,\;i}={\left[1+{\tilde{\gamma }}_{ii}(e^{\tilde{\eta }d_{k}}-1)\right]}^{-1}$ for the current values ${\tilde{\gamma }}_{ij}$ and $\tilde{\eta }$ of γ_*ij*_ and η. Also the argument function for η becomes

(11) $$v(\eta )=\sum_{k=2}^{M}[\sum_{i,\;j}\log\left(1-e^{-\eta d_{k}}\right)\;\kappa_{k}(i,\;j)-\sum_{i}\kappa_{k}(i,\;i)\{(1-{\tilde{\zeta }}_{k,\;i})\log(1-e^{-\eta d_{k}})-{\tilde{\zeta }}_{k,\;i}d_{k}\eta \}].$$

Then, the maximizer γ^*m*^ and η^*m*^ in MM algorithm can be obtained as follows.

**Step 0:** Set initial parameters ${\tilde{\gamma }}_{ij}=\gamma_{ij}^{\ell }$, and $\tilde{\eta }=\eta^{\ell }$.

**Step 1:** Update ${\tilde{\gamma }}_{ij}$ by maximizing Equation (10), that is,

γijm=∑k=2Mκk(i, j)/∑k=2M[∑j′κk(i, j′)−ζ˜k, iκk(i, i)]  for i=jγiim=1−∑j=iγijm.       for i=0, 1, 2, 3, and 4.

**Step 2:** Find new $\tilde{\eta }$ by maximizing the argument function (Equation 11) using the Newton–Raphson algorithm.

**Step 3:** Repeat Steps 1 and 2 until ${\tilde{\gamma }}_{ij}$ and $\tilde{\eta }$ are converged.

The convergent parameters are the maximizer of the second part in Equation (7).
